# Interventions to Reduce Stigma Related to Mental Illnesses in Educational Institutes: a Systematic Review

**DOI:** 10.1007/s11126-020-09751-4

**Published:** 2020-05-05

**Authors:** Ahmed Waqas, Salma Malik, Ania Fida, Noureen Abbas, Nadeem Mian, Sannihitha Miryala, Afshan Naz Amray, Zunairah Shah, Sadiq Naveed

**Affiliations:** 1 Institute of Population Health Sciences, University of Liverpool, Liverpool, UK; 2 Program Director: Child and Adolescent Psychiatry Fellowship, Institute of Living/Hartford Healthcare, Hartford, CT USA; 3 King Edward Medical University, Lahore, Pakistan; 4 FMH College of Medicine & Dentistry, Lahore, Pakistan; 5 Mental Health Counselor, PICACS, Washington, USA; 6 Integrated Psychiatric Consultants, Kansas, KS USA; 7 Dow University of Health Sciences, Karachi, Pakistan; 8 Weiss Memorial Hospital, Illinois, Chicago, USA; 9 Psychiatry and Behavioral Sciences, University of Kansas Medical Center, Kansas, USA

**Keywords:** Stigma, Institutions, Mental health, Depression, Psychosis, Anxiety, Autism, Interventions

## Abstract

This investigation reviews the effectiveness of anti-stigma interventions employed at educational institutes; to improve knowledge, attitude and beliefs regarding mental health disorders among students. Preferred Reporting Items for Systematic Reviews and Meta-Analysis (PRISMA) checklist guidelines were followed and protocol was registered in PROSPERO (CRD42018114535). Forty four randomized controlled trials were considered eligible after screening of 104 full-text articles against inclusion and exclusion criteria.

Several interventions have been employed to tackle stigma toward psychiatric illnesses, including education through lectures and case scenarios, contact-based interventions, and role-plays as strategies to address stigma towards mental illnesses. A high proportion of trials noted that there was a significant improvement for stigma (19/25, 76%), attitude (8/11, 72%), helping-seeking (8/11, 72%), knowledge of mental health including recognition of depression (11/14, 78%), and social distance (4/7, 57%). These interventions also helped in reducing both public and self-stigma. Majority of the studies showed that the anti-stigma interventions were successful in improving mental health literacy, attitude and beliefs towards mental health illnesses.

## Introduction

Mental health disorders are prevalent worldwide, with detrimental personal, social and financial consequences [1]. It is estimated that adult mental and substance disorders account for 7% of all global burden of diseases and 19% of all years lived with disability [2]. Overall, mental health illnesses account for 16% of the global burden of disease and injury in people aged 10–19 years, with suicide being the third leading cause of deaths in adolescence [2]. Adolescents with behavioral disorders are particularly vulnerable to social exclusion and stigma, educational difficulties, overall poor health and risk-taking behaviors (e.g. sexual risk taking, substance use and aggression) [3]. Despite their presentation as a major public health concern, these conditions often go undetected and untreated. And individuals with mental health disorders report distress and poor access to healthcare due to fear of stigma, prejudice and discrimination in the society [3]. Among adolescents especially, the prevalent stigma in the educational setting, can exacerbate loneliness and isolation, often associated with suicidal behaviors [3].

Stigma, in general, is conceptualized as a feeling of disgrace, shame, and self-blame that results in social exclusion, isolation, and embarrassment [4]. Elliot et al., report that *branding* of individuals with mental illnesses is often associated with deviance, dangerousness and social illegitimacy [5]. These individuals, therefore, experience “label avoidance” restricting help-seeking and fearing negative reactions from others [4]. These stigmatizing perceptions toward individuals with mental illnesses can manifest in discriminatory forms such as withholding access to care, coercive treatment, avoidance, and segregated institutions (*structural stigma*) [6]. Thus, these individuals are burdened by the distress of their symptoms and the distress of the stigma.

The stigma is often divided in two forms; public stigma and self-stigma [6]. Public stigma is described as the attitude and reaction of general population towards people with mental illnesses while self-stigma corresponds to the internalized shame, guilt and poor self-image caused by acceptance of the societal prejudice [6]. Unfortunately, stigma towards mental illnesses is prevalent among all strata of our society including medical professionals [6]. This stigma is often aggravated by the stereotypical and prejudiced portrayal of mental illnesses in the media. Empirical investigations on media reporting suggest that individuals with mental illnesses are shown as deviants: “homicidal maniacs”, weaker individuals, and one with childlike perceptions [6]. Mental health illnesses and associated stigma lead to a vicious cycle resulting in poor access to mental and physical healthcare, decreased life expectancy, social exclusion in form of academic termination, unemployment, poverty, homelessness, and contact with criminal justice systems [7].

The issue of stigma toward mental illnesses is even more complex among adolescents- According to De-Luca, research in this domain is scarce (accounting for 3% of research) [8]. It is important to understand it among youth, delineate processes and barriers especially mental health attitudes and knowledge and help-seeking behaviors [8]. This is especially important because adolescence is a crucial period in an individual’s psychosocial and emotional development. At this age, the need for peer approval and inclusive social networks dictate how an adolescent cope with the double burden of mental health problems and rejection from classmates [8]. Understanding the dynamics of stigma and effects of peer perception in educational settings on identity development of the youth with mental illnesses is particularly important. This has been found to be a significant barrier in over 68% of the countries globally in a survey conducted by the World Health Organization (WHO) [8].

The WHO explicitly recommends developing programs to improve stigma and psychiatric outcomes. Recent reviews examining anti-stigma interventions in high-income countries, have shown short-term improvement in knowledge, awareness and in attitude towards mental health illnesses [9]. Studies measuring long-term effectiveness (beyond four weeks) suggested improvement in attitude and knowledge but these benefits could not translate into improvement in behavioral outcomes [9]. However, there have been fewer to no evidence synthesis efforts for educational institute -based interventions especially in the low- and middle-income countries (LMIC). Therefore, to address this paucity of data, the present review aims to summarize evidence pertaining to *anti-stigma interventions* for mental illnesses in educational institutes.

## Methods

This systematic review follows the guidelines of the Preferred Reporting Items for Systematic Reviews and Meta-Analysis (PRISMA) checklist [10]. Its protocol was registered apriori in PROSPERO (CRD42018114535).

### Operational Definitions

Using the framework of consensus study report on *The Evidence for Stigma Change*, we defined public stigma as societal reaction to an individual’s mental illness [11]. We included evidence pertaining to all the societal groups irrespective of education, socioeconomic strata or occupation. Self-stigma was defined as internalized feelings of shame, guilt and worthlessness in reaction to societal stigma [11].

### Search Process

To gain an understanding of these interventions in a broader scope, we did not limit ourselves to specific psychiatric diagnoses, and made use of general search terms pertaining to psychiatric illnesses. However, we also included several terms pertaining to common mental disorders among adults and pediatric population to ensure none of the disorders important in the context of global mental health are missed. Eight academic databases including CINAHL, PubMed, Cochrane Library, Global Health Library, Virtual Health Library, POPLINE, Psycarticles, and Psycinfo and Web of Science, were searched on September 17th, 2018, using search terms noted in Table 1. No restrictions or database filters were applied regarding language, time period or publication year. The database search was also augmented by manual searching of bibliography of eligible studies.

**Table 1:** Search Term Used

**Concept**	**Keywords**
*Stigma*	Stigma OR stigmas OR stigmatization OR stigmatization OR “Social Stigma”[MeSH]
*Psychiatric diagnoses*	mental OR Psychiatr* OR Psychological OR depress* OR anxiety OR anxious OR suicid* OR psychosis OR schizophreni* OR trauma* OR attention-deficit OR hyperactivity OR oppositional-defiant OR autism OR “disruptive mood dysregulation disorder”
*Study design*	intervention OR trial OR RCT OR randomized-controlled
*Setting*	school* OR institut* OR college* OR universit*

### Study Selection

After automated removal of duplicates from bibliographic records using Endnote, we scrutinized their titles and abstracts against our pre-specified inclusion and exclusion criteria. The full texts of eligible titles identified in this phase were further scrutinized against the eligibility criteria. This phase was performed by two reviewers working independently from one another, under supervision of a senior reviewer.

We included all randomized controlled trials (RCTs) assessing the effectiveness of interventions or campaigns in educational institutions (schools, colleges, universities), that were primarily aimed to reduce stigma related to psychiatric disorders. No restriction of age, language, race, gender, ethnicity, geographic location, publication year will be applied. We did not consider any interventions which were not conducted in context of academic institutions.

### Data Extraction & Analysis

Data extraction pertaining to eligible studies was performed using a standardized template by one reviewer, including bibliographic details, institutional and regional affiliations, characteristics of the study sample, and characteristics of interventions. Characteristics of study sample included the characteristics of the population of interest, age and geographical scope. While the characteristics of intervention focused on the targeted diagnosis, names of scales utilized to assess stigmatizing attitudes toward mental illnesses, and the primary outcomes measured. The interventions were stratified in three groups, according to their deliver agents: medical doctors, nurses, and psychology professionals. We also classified the interventions according to their theoretical orientations and noted the content of interventions. Later, a careful analysis of the theoretical orientation and content of interventions by the senior authors, based on an adapted version of matching and distillation framework. This enabled us to *unpack* these interventions into common elements or strategies employed.

Risk of bias in the studies was assessed using the Cochrane’s tool for assessment of risk of bias in RCTs across several matrices: a) randomization procedure b) allocation concealment c) blinding of participants and personnel d) blinding of outcome assessors e) attrition bias f) Other biases. Data extraction and quality assessment were performed by two independent reviewers and any disagreement were resolved by discussion or the guidance of senior reviewer. Unfortunately, due to methodological and statistical heterogeneity, we deferred the application of meta-analysis, to yield the pooled effectiveness of these interventions in reduction of stigma towards mental illnesses.

## Results

**Fig. 1:**
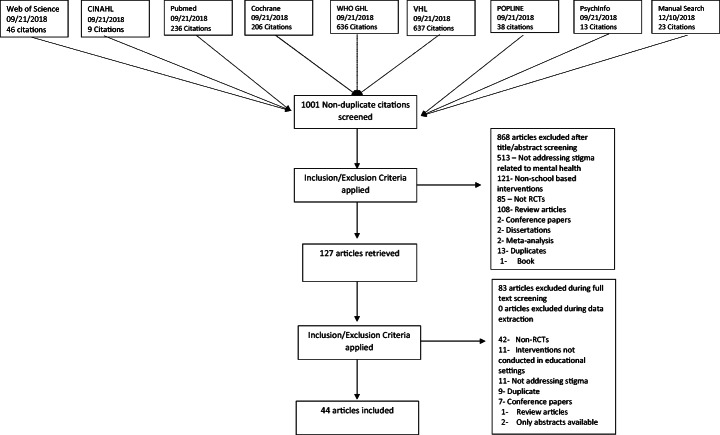
PRISMA Flow Diagram

Our academic searches yielded a total number of 978 non-duplicate references, which were screened for eligibility based on their titles and abstracts. Out of these, 104 full texts were retained after exclusion of 868 citations. Thereafter, 44 RCTs s were deemed eligible after a careful review of their full texts, against the inclusion and exclusion criteria set apriori. Detailed results have been presented in PRISMA flow diagram (Fig. 1).

### Study Characteristics

The eligible publications were published between the years 1998 and 2018. A majority of these interventions were conducted in high income countries and regions including USA (*n* = 15), Australia (*n* = 7), Greece (*n* = 4), UK (*n* = 3), Germany (n = 1), Canada (n = 1), Portugal (n = 1), Taiwan (n = 1), Hong Kong (n = 1), Spain (n = 1), Japan (*n* = 2), and Korea (n = 1). While only 6 studies were conducted in upper and lower middle-income countries including China (n = 2), Russia (*n* = 2) Nigeria (n = 1) and Brazil (n = 1). Low income countries did not contribute to any RCTs in this domain.

### Setting & Delivery of Interventions

Most of the evidence was from RCTs conducted in the context of urban settings (*n* = 27), followed by mixed settings (*n* = 5), suburban (n = 4), and rural (n = 1). The geographical region was unspecified in seven studies. A higher proportion of interventions (*n* = 20) were conducted in school settings (primary school, secondary schools, high school). This was followed by graduate schools/university setting (*n* = 6), undergraduate and graduate students enrolled in psychology courses (n = 5), non-psychology undergraduate setting (*n* = 3), and adult schools (n = 2). Six studies were conducted in medical schools (n = 3) and nursing schools (n = 3). Among these studies, 25 were conducted in adults or predominantly adult population, 18 in adolescents or predominantly adolescent population, and one in children. The age ranged varied widely with lowest mean age of 13 years [12] and highest was 43.1 years [13].

### Quality Rating

**Fig. 2:**
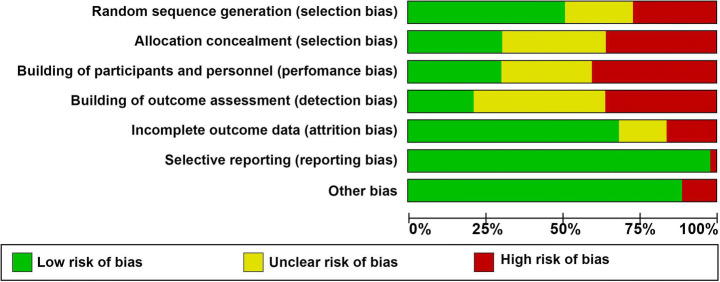
Risk of Bias Graph

**Fig. 3:**
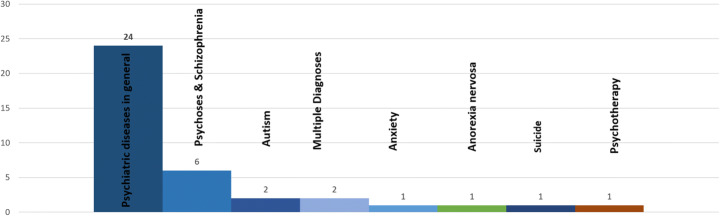
Mental health conditions targeted in stigma reduction interventions

Random sequence generation was at a high/unclear risk of bias among 22 trials and allocation concealment (29 RCTs). Frequencies of studies reporting a high risk/unclear across other domains of Cochrane risk of bias tool were: blinding of outcome assessors (*n* = 35), blinding of participants and personnel (*n* = 31), attrition bias (*n* = 14), other sources of bias (*n* = 5), and selective reporting (*n* = 1). A total of 35 studies were rated as having a high risk of overall bias i.e. ≥ 3 matrices of risk of bias tool were rated as having unclear or high risk of bias for these studies (Figs. 2 and 3). Figure 2 presents a clustered bar chart exhibiting frequencies of high, unclear and low risk bias across all matrices of Cochrane risk of bias tool.

### Mental Health Conditions

Mental health illnesses, in general, were the target of these interventions in 24 studies whereas six studies targeted depression. Other targeted diagnoses were psychosis and schizophrenia (*n* = 6), autism spectrum disorder (*n* = 2), anxious-ambivalent attachment (*n* = 1), anorexia nervosa (n = 1), and suicide (n = 1). One study focused on both depression and Tourette syndrome whereas one study addressed depression and schizophrenia. One intervention addressed engagement in psychotherapy treatment. A summary of mental health condition targeted is mentioned in fig. 3.

### Intervention Characteristics

Delivery agents of interventions included researchers (*n* = 17), specialist psychiatrists, and psychologists (*n* = 7), trained mental health professionals (n = 6), school teachers/course instructors (*n* = 4), graduate students (*n* = 2), and peers with lived experiences (n = 2) and researchers and teachers as delivery agent (n = 1). This information was missing for five studies. Numbers of sessions ranged from 1 to 8 sessions where a high proportion of interventions (*n* = 25) were delivered in only one session. The numbers of sessions were unspecified in seven studies. Studies delivered the following number of interventions: two sessions(n = 4), three session (n = 4), four sessions (n = 1) and six sessions (n = 2) and eight sessions (n = 1). The duration for whole program was categorized into studies with duration of one day (*n* = 11), one day to one week (*n* = 8), one to four weeks (*n* = 3), and longer than one month (*n* = 20). The duration was not mentioned in one study [14] while another study had mixed duration depending on the type of intervention employed [15]. The longest duration of an intervention was 48 weeks [16]. The duration for each session varied from 20 min to 12 h.

### Strategies & Elements of Interventions

**Fig. 4:**
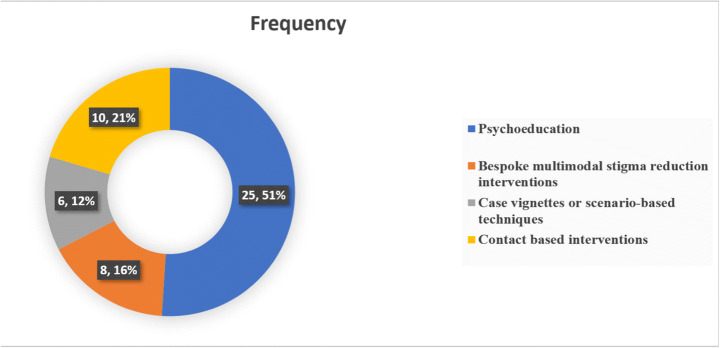
Summary of strategies employed in stigma reduction interventions

These stigma reduction interventions constituted several different strategies as summarized in fig. 4, most common of which were psychoeducation through lectures and discussion with mental health professionals and use of case vignettes and scenario-based interventions. Psychoeducation was also delivered via online platforms including website messages, video-based instructions, and educational short messaging service (SMS). Another important strategy highlighted in this review was contact-based learning where two most important intervention elements were role play and contact-based learning with individuals struggling with mental illnesses. The included interventions used either one teaching method or a mix of the above-mentioned.

### Psychoeducation

Psychoeducation was employed in 25 studies using a variety of delivery techniques, mostly based on etiological models of psychiatric illnesses. These interventions educated participants about different attributes of mental health disorders such as epidemiological factors, clinical features, course of illnesses, and available treatment options. They were delivered through didactic lectures [17–24], photographic images of billboard messages [14], short educational messages [25, 26], video messages [27], structured courses and workshops for students [18–28], and distribution of booklets and slideshows [29]. Some of authors utilized a multimodal approach for delivering their interventions, for instance, Papish et al. (2013) structured a course on different mental illnesses using teaching methods such as didactic teaching, case-based teaching with group discussions, and an optional movie night [28]. Pereira and colleagues (2014) used a mixed approach by using tutorials, videos, interactive discussion, web conference, and written text support to educate participants [I3]. A few of these interventions provided an overview of etiology of mental health disorders; biogenetic, biochemical, neurobiological, biopsychosocial, and contextual factors. Banntanye et al. [17] formulated an educational intervention based on genetic and heritability of mental health disorders as well as a biopsychosocial model involving a complex interplay of biological, social, and psychological factors [17]. Two more models were part of several interventions, for instance, the contextual model linked complicated life situations with etiology of mental health disorders and biomedical factors where neurochemical changes in the brain were studied as a cause of psychiatric disorders [30]. Using a similar model, Han and colleagues presented neurobiological factors as a cause of mental health disorders, in their interventions [19].

### Bespoke Multimodal Stigma Reduction Interventions

Three studies used *Mental Health First Aid (MHFA)* [31–33] as a structured training module to address risk factors and clinical features of common mental health disorders including depression, anxiety, substance use disorders, psychosis, and eating disorders. This intervention program also educated participants on strategies to assist someone experiencing mental health crises. Two interventions tested the effectiveness of health education tools, the *fotonovlea or Secret Feelings* that addressed misconceptions and stigmatizing attitudes through posed photographs, captions, and soap opera narratives [34, 35]. *The Same or Not Same intervention* focused on education about schizophrenia followed by an opportunity to contact with individuals struggling with schizophrenia. In the *Video-Education* Intervention, a video was followed by educational message. In this intervention, there was information about the cause, timeline, course of illness, and different myths [36]. In a classroom-based intervention used in two studies, projective cards were used to understand misperceptions about mental illnesses and discussion to overcome these misperceptions was carried out. In last part, patient’s narratives and role of media were also added [37, 38].

*Case vignettes or scenario-based techniques* were employed in six interventions to enhanced understanding of different aspects of mental health disorders [15, 39–43]. Mann et al. evaluated change in stigmatizing attitudes by comparing scenario-based teaching to provide an opportunity to read education material on mental illnesses [15]. Similar intervention was carried out by using case vignettes [40] and documentary film [42] to address stigma. In another intervention, participants were delivered a lecture on schizophrenia whereas second group had a scenario-based activity of four individuals with schizophrenia in remission [39]. It highlights the living arrangements, daily activities, needs, interests and social support system of individuals with schizophrenia [39]. Nam and colleagues (2015) used documentary to create stigma manipulating scenarios among college students with anxious-ambivalent attachment [42]. In another study, the intervention group received didactic lectures regarding factual knowledge about mental health and illness followed by case vignettes. The myths associated with mental illness, positive attitudes toward persons with mental illness, and resources to receive mental health care were examined during a group activity [43].

*Contact-based interventions* were assessed in 10 studies by using direct interaction with patients [12, 44–50] and filmed or video techniques [12, 45, 48, 51, 52]. In an intervention, two service users on DVD described personal view of mental health and stigma followed by fact-based experience in nine key areas related to mental health disorders [45]. In Live intervention group, this exercise was conducted in live sessions whereas the control interventions delivered information about mental health and related stigma through a lecture [45]. *In Our Own Voice (IOOV)*, two group facilitators with history of mental health disorders addressed five components including “Dark Days, Acceptance, Treatment, Coping Mechanism, and Success. It also had corresponding videotaped sessions for each component [46, 47]. The *eBridge intervention* was structured on personalized feedback about symptoms in individuals with history of suicidal behaviors along with access to resources based on the principles of motivational interviewing [53]. Self-affirmation psychotherapy was provided in a study by Lannin and colleagues [54]. In this program, an individual with mental illness is advised to repeat a positive statement or set of such statements about the self on a regular basis to inspire positive view of the self and reduce negative thinking, or low self-esteem [54]. Chisholm and colleagues (2016) work especially among adolescents concluded that educational interventions provide far more promising results than contact-based interventions [44]. Although not consistent with previous literature reporting this comparison [44], Chrisholm et al., argue for a different teaching approach for adolescents keeping in view their level of maturation, influence of the media, and that the information processing and understanding of mental illnesses differ among adolescents, as proposed in several conceptual frameworks [44].

### Outcomes

**Fig. 5:**
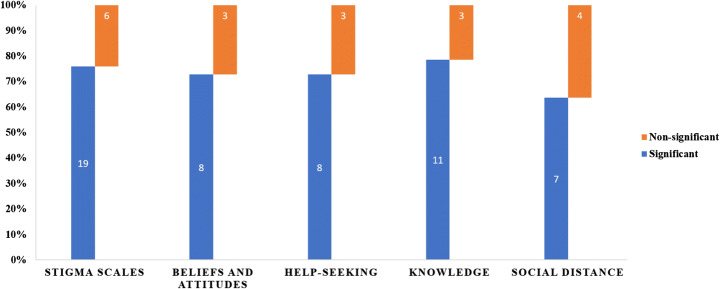
Proportion of studies demonstrating reduction in stigma related outcomes

The eligible studies reported effectiveness of these intervention on a heterogeneous body of scales, which measured stigma toward psychiatric illnesses, pre and post knowledge among participants, attitudinal and intentional changes, and recognition of psychiatric symptomatology. Moreover, help-seeking practices were also measured in these interventions. A lot of importance was placed on public stigma rather than self-stigma. The most frequent outcome was stigma (*n* = 25), followed by changes in knowledge levels (n = 25), attitude (*n* = 11), help-seeking (n = 11), social distance (*n* = 9), and recognition and literacy regarding depression (*n* = 5). Majority of studies reported an improvement in stigmatizing attitudes towards mental health disorders with improvement in both public and self-stigma (Fig. 5).

### Stigma

Among 25 studies addressing stigma, where a higher proportion (*n* = 19) studies reported a significant improvement in stigma levels at the study endpoint. In a study, comparing biogenetic intervention with a multifactorial intervention, there was no difference among both intervention groups from baseline line to study endpoint [17]. However, there was a significant improvement among these intervention groups from the control group. While deciphering between personal and public stigma, five studies reported stigma-related outcomes for these two concepts (personal = 3, public = 2) with only one study showing non-significant improvement [33]. Another study saw improvement at the first timepoint, but it failed to last until second visit. Out of five studies showing non-significant or no improvement, three reported non-significant change [14, 26, 40] whereas two reported no change from baseline to study endpoint [23, 47].

The study on biological anti stigma intervention by Boucher and colleagues (2014) attributed non-significant improvement in stigma scores to the conceptualization of depression in as a brain disease whereas depression results from various biolopsychological factors [14]. Two studies using psychoeducation as a tool of change pointed out that lack of significant improvement in stigma is possibly due to sole use of educational interventions [23, 26]. Pinto-Foltz and colleagues had a smaller sample size resulting in the lack of significant improvement in stigma scores [47].

### Beliefs and Attitudes

The attitude was reported in 11 studies with improvement in eight studies at the study endpoint compared to baseline. One study reported improvement in authoritarianism and social restrictiveness (sub-scales of Community Attitudes Toward Mental Illness scale) while benevolence and community mental health ideology subscales failed to show significant improvements [42]. In remaining three studies, there was non-significant or lack of improvement. The duration of program has limited impact on the favorable results as one intervention had duration of three days [43]. The two other studies had duration of one [24] and eight weeks [32]. Winkle et al. (2017) reported small effect size for flyer or control group and medium effect size for the experimental group [22]. After a psychiatry clerkship, medical students reported improvement in stigmatizing attitudes but there was no improvement in attributions regarding responsibility and readiness to provide care to patients with mental illnesses [50]. Moreover, the lack of educational intervention may not be sufficient to change beliefs [26, 36]. This underscores the need to strengthen medical school clerkships as well as enhancing the ways to interact with this patient population.

### Help-Seeking

Intention and attitude to seek help were reported in 11 studies with improvement in eight studies. One study reported similar improvement among the intervention and control groups because the control group also received psychoeducation through brochures on depression [35]. In remaining three studies, there was non-significant or lack of improvement post-intervention [23]. In a rural-area based study, there was improvement in stigma and attitudes but it failed to materialize this change into help-seeking behaviors [23]. It is worth noting that adolescents in rural areas were more likely to turn to family and friends than seeking help from professionals such as school counselors [23]. In another study, the lack of improvement was regarded to inadequate dosage or duration of the program among adolescents [29]. The causation of depression as “neurological disease” was also a barrier and may have prevented college students from seeking help [14].

### Knowledge of Mental Health Disorders and Treatment

The knowledge of mental health disorder and treatment was assessed in nine studies and depression literacy in five studies. Out of these 14 studies, 11 reported significant improvement in help-seeking with no improvement in three studies. It is interesting to note that two studies reported improvement in knowledge regarding depressive disorders and relevant treatment but engagement in these treatments was non-satisfactory, with a drop rate exceeding 44% [23, 29]. The lack of improvement in three studies was reported due to lack of past experience or contact with individuals with mental illnesses [33, 47]. In study by Pinto-Foltz, the improvement was more noticeable at 4 and 8-week timepoints among intervention group, owing to past exposure of participants in control group to mental health information [4]. For *Depression Fotonovela* intervention, lack of difference in improvement among both experimental and control groups was due to ceiling effect and higher baseline knowledge scores [34].

### Social Distance

The social distance was assessed in seven studies with four studies reporting favorable results. Majority of the studies with favorable results had two important components including opportunity to contact with individuals with mental illness (*n* = 3) and participants with a background in medicine or nursing (*n* = 4). In one of these studies, a combination of two education strategies didactic education and video group fared better than the education group alone. Three of the studies reported a non-significant outcome; these studies lacked contact with individuals diagnosed with mental health disorders. Moreover, participants in these interventions were adult individuals with no background in relevant professions [34], middle school students, or students enrolled in psychology courses [55].

## Discussion

This article reviews the evidence for various aspects of stigma towards mental health disorders in educational settings. These interventions were carried out in university, college, and school settings, targeting a wide range of mental health disorders. Duration of intervention varied widely with most of the interventions lasting for more than four weeks. For outcome measure, majority of studies reported significant improvement for stigma (19/25, 76%), attitude (8/11, 72%), help-seeking (8/11, 72%), knowledge of mental health including recognition of depression (11/14, 78%), and social distance (4/7, 57%). It is worthwhile to appreciate that all these outcomes measure are intercalated and have a directional effect on each other.

Most of the studies included in this review focused on reduction of public stigma rather than self-stigma, two different yet highly intercalated concepts. The reviewed interventions targeted one or more of the core stigmatizing behaviors especially fear and exclusion and authoritarianism that people with mental illnesses face and inspire benevolence and compassion among the intervention recipients [56]. This was done through different strategies, most frequently being psychoeducation through didactic lectures. Other strategies were contact-based interventions, and role-plays to address stigma towards mental illnesses. Reduction of self-stigma was done through a specialized program of self-affirmation therapy, to inspire moral and adaptive adequacy of the self, with a main aim to inspire positive view of self [54].

All of these interventions followed the recommendations proposed by Corrigan et al., who recommended three ways to combat public stigma: protest, education, and contact to combat the existing stigma [56]. The latter approaches were employed frequently, however, protest in response to the stigmatizing environment propagated by public statements, media reports, and advertisements was absent in these initiatives [56]. In addition, it is also imperative to engage health care providers, stakeholders, policymakers, for development of campus-based policies combating stigma [57]. We did not find any interventions designed at the policy level as well as the system level or reporting their effectiveness and wide-ranging implications and socioeconomic benefits.

A plethora of research in recent decades has shown that stigmatizing attitudes toward mental illnesses are strongly driven by sociocultural and religious factors, as well as individual factors especially empathy and experience and education levels [58–61]. This is particularly relevant in context of low- and middle-income countries where such attitudes and beliefs toward mental illnesses are prevalent even among the learned. For instance, the belief in djinni possession, black magic and divine punishment as causes of mental illnesses are rampant in the Indian subcontinent. This requires the development of very culture specific interventions in these countries. And yet only one of the interventions targeting stigma toward mental illnesses has been developed in these countries [62]. This is a major gap that must be addressed by development of interventions that aim to mitigate negative cultural and social norms as well as inspire benevolence toward people with mental illnesses. It can be argued that development, testing and implementation of relevant interventions in poorer nations can foster an alternative and correct view of mental illness resulting in improved knowledge and linkage to services.

This systematic review has several strengths. An electronic search of academic databases combined with manual searching for references provides an exhaustive search for relevant evidence. It provides an overview of RCTs of interventions targeting stigma towards mental health in educational settings as well as the strategies used in each intervention and different components of these interventions. Combined with a qualitative assessment of the theoretical orientation of these intervention as well as assessment of risk of bias, makes this review an important source for development and testing of future interventions in this area. However, this review also has several limitations. Due to heterogeneity, varying intervention design, and different outcome measures, meta-analysis could not be performed. It is also important to consider the higher risk of bias in included studies while interpreting the results of these studies. These interventions were important in challenging the stereotypes and prejudice by providing an opportunity of social contact with individuals with mental illnesses, engaging in myth-busting, and increasing awareness of mental illnesses through education via text, lecture, or film [7]. However, due to lack of meta-analytic evidence, it is difficult to ascertain if a single component intervention is any better than its multi-component counterparts such as DVD or direct contact group work better than other. Although, generally a greater improvement was reported with comprehensive approaches to combat stigma [45].

This review provides some promising empirical support for anti-stigma interventions regarding mental health disorders aimed at students. These interventions were somewhat successful in reducing both self and public stigma. This highlights the need for progressively thorough, better-quality evaluations conducted with more diverse samples of the population. As it appears that short-term interventions often only have a transient effect, the implication is that researchers should study longer term interventions and to use the intervening time and outcome data to improve the interventions along the way. Future research should explore to what extent changes in students’ knowledge, attitudes, and beliefs can result in earlier help seeking.
